# Development of a Hierarchical Variable-Number Tandem Repeat Typing Scheme for *Mycobacterium tuberculosis* in China

**DOI:** 10.1371/journal.pone.0089726

**Published:** 2014-02-25

**Authors:** Tao Luo, Chongguang Yang, Yu Pang, Yanlin Zhao, Jian Mei, Qian Gao

**Affiliations:** 1 Key Laboratory of Medical Molecular Virology of Ministries of Education and Health, Institutes of Biomedical Sciences and Institute of Medical Microbiology, School of Basic Medical Sciences, Fudan University, Shanghai, China; 2 Chinese Center for Disease Control and Prevention, and Beijing Tuberculosis and Thoracic Tumor Research Institute, Beijing, China; 3 Department of TB Control, Shanghai Municipal Centers for Disease Control and Prevention, Shanghai, China; St. Petersburg Pasteur Institute, Russian Federation

## Abstract

Molecular typing based on variable-number tandem repeats (VNTR) analysis is a promising tool for identifying transmission of *Mycobacterium tuberculosis*. However, the currently proposed 15- and 24-locus VNTR sets (VNTR-15/24) only have limited resolution and contain too many loci for large-scale typing in high burden countries. To develop an optimal typing scheme in China, we evaluated the resolution and robustness of 25 VNTR loci, using population-based collections of 1362 clinical isolates from six provinces across the country. The resolution of most loci showed considerable variations among regions. By calculating the average resolution of all possible combinations of 20 robust loci, we identified an optimal locus set with a minimum of 9 loci (VNTR-9) that could achieve comparable resolution of the standard VNTR-15. The VNTR-9 had consistently high resolutions in all six regions, and it was highly concordant with VNTR-15 for defining both clustered and unique genotypes. Furthermore, VNTR-9 was phylogenetically informative for classifying lineages/sublineages of *M. tuberculosis*. Three hypervariable loci (HV-3), VNTR 3232, VNTR 3820 and VNTR 4120, were proved important for further differentiating unrelated clustered strains based on VNTR-9. We propose the optimized VNTR-9 as first-line method and the HV-3 as second-line method for molecular typing of *M. tuberculosis* in China and surrounding countries. The development of hierarchical VNTR typing methods that can achieve high resolution with a small number of loci could be suitable for molecular epidemiology study in other high burden countries.

## Introduction

Tuberculosis (TB) remains a serious global public health issue, especially in the developing world. Over 95% cases and deaths caused by TB were in developing countries [1]. China has been suffering the second largest TB burden worldwide. In 2011, there were about one million new cases in China, which accounted for 12% of the global epidemic [1]. The TB epidemic in China was exacerbated by the dominance of the notorious *M. tuberculosis* Beijing strains [2], as well as the prevalence of multidrug resistant (MDR) cases [3]. Both the Beijing strains and MDR strains in China were found associate with ongoing transmissions [3], [4]. Therefore, there is an urgent need for reliable genotyping tools to identify and prevent transmissions of *M. tuberculosis*.

Among the genotyping tools, the PCR-based variable-number tandem repeat (VNTR) analysis represented a promising method for typing *M. tuberculosis* [5], [6]. The newly proposed 15- or 24-locus VNTR typing sets (VNTR-15/24) have demonstrated adequate discriminatory power for tracing transmissions in low-burden areas [7]–[10]. However, their usefulness in high-burden settings was questioned, especially in settings such as China, where Beijing strains were prevalent [11]–[13]. *M. tuberculosis* Beijing strains are genetically highly similar, which leads to limited discriminatory power of VNTR-15/24 in settings dominated by these strains [13]–[15]. Several locally optimized VNTR schemes, which include the hypervariable loci such as VNTR 3232, VNTR 3820 or VNTR 4120, were suggested for typing Beijing strains [15]–[18]. However, hypervariable loci have defects, such as amplification failures and uninterpretable large amplicons [5], [14]. Therefore, these schemes are less applicable as standard typing methods. Using hypervariable loci as second-line method to subtype clustered strains following the VNTR-15/24 represents a more appropriate solution [13], [14]. Recently, a consensus set of four hypervariable loci was proposed for second-line typing of Beijing strains following the VNTR-24 [19]. However, the VNTR-15/24 contains too many loci to be extensively applied in China, which has limited resources but a huge TB burden. Furthermore, a number of loci from VNTR-15/24 have been demonstrated poor resolutions in Beijing strains [12], [15], [18], [20].

In this study, we evaluated the discriminatory power and robustness of 25 VNTR loci using population-based collections of *M. tuberculosis* isolates from six provinces in China. Our aim was to develop an optimal VNTR scheme as potential standard for typing *M. tuberculosis* in China.

## Materials and Methods

### Selection of Candidate VNTR Loci

According to the published data in different regions of China and surrounding countries [12], [15]–[18], [21]–[28], we summarized the Hunter-Gaston index (HGI) of 37 VNTR loci and calculated the median for each locus (Table S1). According to Sola et al. [29], the discriminatory powers of VNTR loci could be classified as high (HGI>0.6), moderate (0.3≤HGI≤0.6) and low (HGI<0.3) levels. When this standard was applied to HGI medians of the 37 loci, 19 of them were found with low discriminatory power. To include as many potentially discriminatory loci as possible, we set a threshold of ≥0.1 for choosing candidate loci in this study. Loci that belong to standard VNTR-15 were all included. At last, 27 VNTR loci were selected for evaluation in this study.

### Clinical Isolates and VNTR Typing

Population-based collections of 1375 isolates from six provinces (Guangxi, 176; Sichuan, 216; Shanghai, 396; Shandong, 206; Henan, 197; Heilongjiang, 184) were used to evaluate the candidate VNTR loci. Genomic DNAs of all isolates were extracted with a boiling lysis method, and were typed with a 16-locus VNTR set in our previous study [4]. Additional typing of 11 loci was performed in this study using the primers listed in Table S2. The PCR reactions for 22 loci were performed in a volume of 10 µL containing 1× Taq PCR MasterMix (CoWin Biotech Co. Ltd., Beijing, China), 0.4 µM of each primer, and 1 µL DNA template. The reactions for the remaining 5 loci (VNTR 3232, VNTR 3820, VNTR 4120, VNTR 3336 and QUB-15) were performed in a volume of 20 µL containing 1× GC buffer I (Takara Biotech Co. Ltd., Dalian, China), 200 µM of each dNTP, 0.5 U of Taq (Takara Biotech Co. Ltd.), 0.4 µM of each primer, and 1 µL DNA template. The thermocycling conditions were as follows: 95°C for 5 min, followed by 30 cycles at 94°C for 30 s, 58°C (64°C for locus ETR-F, QUB-1895, QUB-3232, MIRU 40, VNTR 4120) for 30 s, 72°C for 30 s (1.5 min for locus VNTR 3232, VNTR 3820, VNTR 4120 and VNTR 3336), with a final extension at 72°C for 7 min. The size of amplicons were analyzed on 1.0% or 1.5% agarose gels for 1.5 to 2 hours at 150 V with 50 bp Ladder and 100 bp High Ladder size standards (CoWin Biotech Co. Ltd., Beijing, China). Beijing strains and sublineages were identified by RD105 targeted multiplex PCR and the typing of six single nucleotide polymorphism loci in the previous study [4].

A second set of 69 strains that serially isolated from 31 patients were typed with all candidate loci. The comcordance of VNTR alleles of the serial isoaltes from the same patients was used to evaluat the clonal stability of each VNTR locus.

### Allelic Variability and Genetic Distance Analysis

Custom Perl script A was written to calculate HGI of individual VNTR locus in different geographical/genetic populations according to the equation derived from elementary probability [30]. Perl script B was written to calculate the HGI for all possible combinations of the candidate VNTR loci. BioNumerics (version 5.0, Applied Maths, Sint-Martens-Latem, Belgium) was used to construct the Minimal Spanning Trees (MSTs) based on VNTR data. The priority rules was set to first link types that had the highest number of single-locus variants (SLVs). Creation of hypothetical types was not allowed. Creation of clonal complexes was defined by setting the maximum number of variations to fewer than two loci, for more than two genotypes. The frequencies of SLVs, double-locus variants (DLVs) and triple-locus (TLVs) for different VNTR loci were calculated according to the MSTs.

## Results

### Robustness and Variability of VNTR Loci

**Figure 1:**
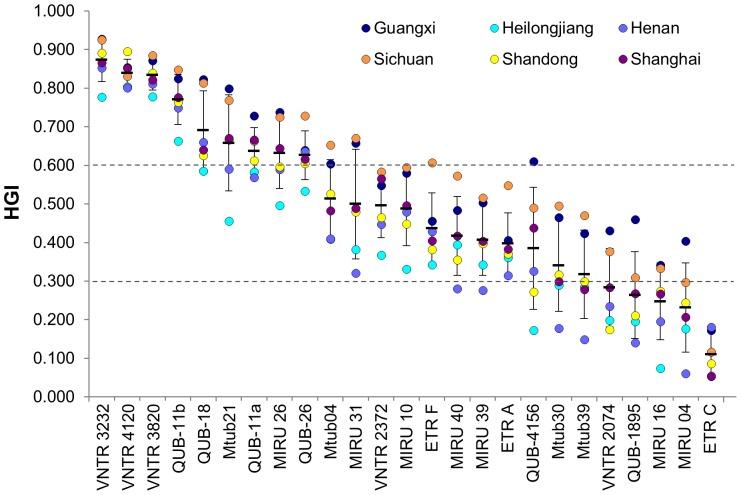
The discriminatory powers of 25 VNTR loci in strains from six field sites. Two dotted lines indicated the thresholds for defining high (HGI>0.6), intermediate (0.3≤HGI≤0.6) and low (HGI<0.3) discriminatory powers. The mean value and standard deviations for each locus were indicated.

Among the 1375 isolates, 1362 (99.1%) of them had enough DNA extracts for additional VNTR typing in this study. Two loci, QUB-15 and VNTR 3336, cannot be amplified for most DNA extracts, even different primers and conditions were tried (Table S2). These two loci were excluded for further analysis. The variability of the remaining 25 loci was evaluated as HGI in different genetic and geographic *M. tuberculosis* populations. Genetically, the 1362 strains were classified as Beijing and non-Beijing strains, and the Beijing strains were further divided into “ancient” and “modern” group according to the single nucleotide polymorphism (SNP) in codon 58 of *mutT2* (Table S3) [4], [13]. The variability for most loci was higher in non-Beijing strains than in Beijing strains. Among Beijing strains, most loci showed higher variability in “ancient” strains than in “modern” strains. Only 9 of the 25 loci showed high or moderated variability (HGI ≥0.3) in “modern” Beijing strains. By contrast, as many as 15 loci in “ancient” Beijing strains and 23 loci in non-Beijing strains had moderate or high variability (Table 1). Geographically, except three hypervariable loci, the variability of other loci showed considerable variations among six regions (Figure 1). The variability of most loci was highest in Sichuan or Guangxi, and lowest in Henan or Heilongjiang.

**Table 1:** The average discriminatory powers of 25 VNTR loci in six field sites and their resolutions in genetic subpopulations of *M. tuberculosis*.

Locus number	Locus	Alias	Typeability (%)	HGI (mean ± STDEV)^a^				
				All strains (n = 1375)	Non-Beijing strains (n = 344)	Beijing strains (n = 1031)	“ancient” Beijing strains (n = 258)	“modern” Beijing strains (n = 773)
1	2163b	QUB-11b	95.4	**0.771**±0.065	**0.782**±0.067	**0.692**±0.058	**0.714**±0.095	**0.620**±0.022
2	1982	QUB-18	99.5	**0.691**±0.101	**0.723**±0.086	0.574±0.105	**0.641**±0.056	0.381±0.082
3	1955	Mtub21	99.7	**0.658**±0.125	0.576±0.062	0.521±0.133	0.304±0.142	0.324±0.098
4	2996	MIRU 26	99.9	**0.632**±0.091	**0.666**±0.105	0.456±0.082	**0.656**±0.122	0.378±0.081
5	4052	QUB-26	97.4	**0.627**±0.063	**0.821**±0.039	0.538±0.059	**0.727**±0.041	0.410±0.085
6	424	Mtub04	99.9	0.514±0.100	**0.648**±0.052	0.349±0.061	0.506±0.052	0.281±0.075
7	3192	MIRU 31; ETR E	99.9	0.500±0.142	0.338±0.104	0.266±0.090	0.318±0.112	0.254±0.091
8	2372	VNTR 2372	99.8	0.496±0.083	0.390±0.129	0.311±0.099	0.379±0.133	0.279±0.085
9	960	MIRU 10	100	0.488±0.096	0.410±0.220	0.230±0.099	0.275±0.136	0.203±0.092
10	802	MIRU 40	100	0.417±0.102	**0.637**±0.055	0.219±0.058	0.229±0.184	0.217±0.059
11	4348	MIRU 39	100	0.407±0.092	0.086±0.112	0.142±0.034	0.220±0.199	0.117±0.033
12	2165	ETR A	94.3	0.397±0.080	0.493±0.066	0.188±0.038	0.167±0.041	0.196±0.056
13	4156	QUB-4156	99.9	0.385±0.159	0.386±0.178	0.377±0.158	0.529±0.077	0.086±0.080
14	2401	Mtub30	99.9	0.341±0.119	0.324±0.178	0.076±0.020	0.095±0.057	0.063±0.026
15	3690	Mtub39	99.9	0.318±0.115	0.591±0.054	0.151±0.049	0.259±0.100	0.118±0.048
16	2074	Mtub24	99.5	0.283±0.102	0.554±0.101	0.172±0.085	0.245±0.212	0.091±0.042
17	1895	QUB-1895	99.8	0.264±0.112	0.320±0.082	0.262±0.128	0.467±0.157	0.135±0.037
18	1644	MIRU 16	99.9	0.248±0.100	0.518±0.192	0.123±0.049	0.163±0.131	0.085±0.049
19	580	MIRU 04; ETR D	100	0.232±0.116	0.540±0.143	0.069±0.026	0.025±0.033	0.081±0.047
20	577	ETR C	100	0.111±0.056	0.151±0.044	0.100±0.070	0.083±0.101	0.094±0.070
21 (excluded)	3239	ETR F	99.3	0.437±0.092	**0.663**±0.033	0.270±0.048	0.586±0.132	0.126±0.037
22 (excluded)	2163a	QUB-11a	90.0	**0.637**±0.060	0.554±0.147	0.524±0.045	0.578±0.083	0.485±0.040
23 (second-line)	3232	QUB-3232	98.8	**0.873**±0.056	**0.849**±0.053	**0.835**±0.066	**0.904**±0.036	**0.774**±0.055
24 (second-line)	4120	VNTR 4120	98.7	**0.839**±0.035	0.577±0.099	**0.816**±0.051	**0.886**±0.049	**0.764**±0.066
25 (second-line)	3820	VNTR 3820	95.9	**0.834**±0.040	**0.721**±0.094	**0.766**±0.040	**0.815**±0.129	**0.696**±0.031

a value in bold and underline indicates high discriminatory power (HGI>0.6); value in underline indicates intermediate discriminatory power (0.3≤HGI≤0.6).

The robustness of the candidate loci was evaluated as their typeability (defined as the success rate of amplification) and interpretability (defined as the unambiguity for sizing amplicon through agarose gel electrophoresis). The typeability of the 25 VNTR loci ranged from 90.0 to 100% (Table 1). Locus QUB-11a, ETR A and QUB-11b had the lowest typeability (90.0%, 94.3% and 95.4%). Among the 1362 isolates, 45 (3.3%) of them could not be amplified in all three loci, which account for most of the PCR failures in QUB-11b (45/63) and ETR A (45/77). An addition of 91 isolates showed failures in QUB-11a, which leads to a low typeability (90.0%) in this locus. The typeability of the remaining 22 loci was reliable. However, the interpretability of four loci, including ETR F, VNTR 3232, VNTR 3820 and VNTR 4120 was relatively low. For ETR F, 132 strains (9.7%) showed incomplete repeat in this locus. For the three hypervariable loci, there were 860 (63.1%), 855 (62.8%) and 181 (9.7%) strains in VNTR 3232, VNTR 3820 and VNTR 4120, respectively, showed large alleles (with a repeat number larger than 10), which cannot be or hard to be sized unambiguously (Table S4).

At last, we excluded ETR F for further analysis due to the incomplete repeat alleles, as well as its low variability in Beijing strains. Due to the low typeability or low interpretability, locus QUB-11a and three other hypervariable loci were not suitable for firs-line typing. We maintained these four loci as potential candidates for second-line typing by considering their high variability. The remaining 20 loci (VNTR-20) with reliable typeability and interpretability were chosen as candidates for first-line typing.

### Relative Evolution Rate of VNTR Loci

**Figure 2:**
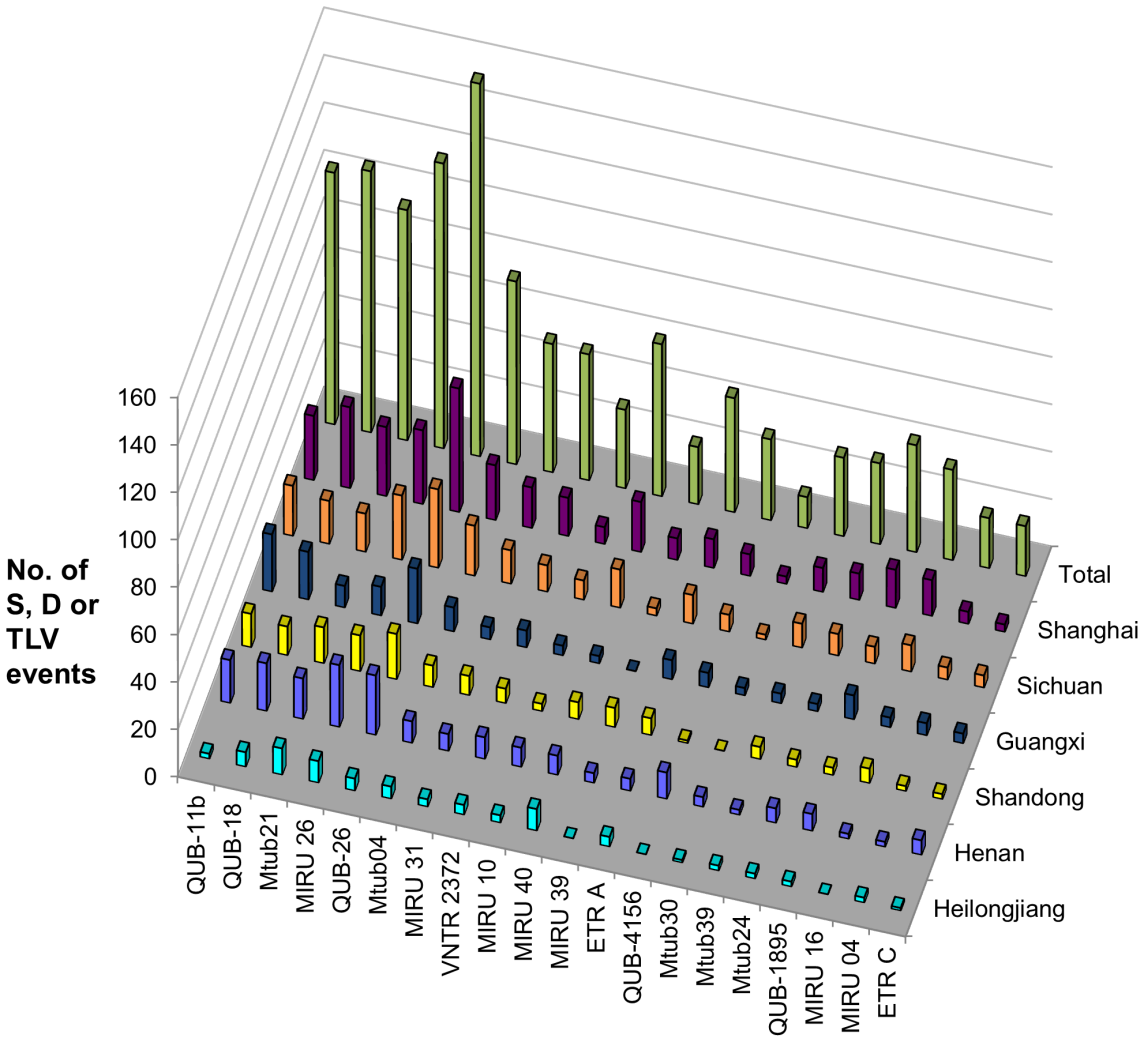
Distribution of single- (S), double- (D), or triple-locus variations (TLV) in 20 robust VNTR loci. Variation events were detected according to the minimal spanning trees of *M. tuberculosis* strains in each field site.

VNTR loci with high variability (as represented by HGI) do not necessarily have high evolutionary rate, as HGI might reflect separate allelic distribution between distinct lineages but not necessarily high variability within lineages [5], [30]. A total of 1167 (85.7%, 1167/1362) strains have unambiguous typing results at all 20 first-line candidate loci. To evaluate the relative evolutionary rate of the 20 loci, we constructed the MSTs for strains of each region based on these loci (data not show). The relative evolutionary rate of each locus was calculated as its involvement in SLVs, DLVs and TLVs according to the MSTs [5]. Comparing to HGI, the consistency of relative evolutionary rates of the 20 loci among six regions were relatively high, indicating similar evolutionary rates (Figure 2). Generally, there was a decreasing trend of evolutionary rate as the HGI decreased. However, several loci, such as MIRU 10, MIRU 39 and Mtub30, showed relatively low evolution rate with respected to their HGIs. To explain the discordance, we analyzed the allelic distribution of the 20 VNTR loci in non-Beijing and Beijing strains (Table S4). Most of the loci showed different allelic patterns between two genetic subpopulations. Therefore, the diversity of these loci in the overall population was partially contributed by variability between subpopulations. Several loci, such as MIRU 39 and Mtub30, showed relatively high variability between subpopulations, but with very low variability within subpopulations, indicating low evolutionary rates.

### Optimization of Locus Set for First-line Typing

Usually, VNTR loci with the highest individual HGIs were selected as the optimal typing set [16]–[18]. However, such procedure ignored the evolutionary rate, as well as the discriminatory redundancy between loci. To avoid these defects, we calculated the discriminatory powers for all possible combinations of VNTR loci in each region. The 1167 strains with unambiguous typing results at all 20 first-line candidate loci were used for the calculation. As we expected, in most cases, the combination with the highest HGI at a given number of locus did not include all loci that had the highest individual HGIs (Table 2).

**Table 2:** The optimal VNTR combinations at different locus number and their average discriminatory powers in all strains or genetic subpopulations of *M. tuberculosis* form six field sites.

No. of loci (no.of possible combinations)	No. of combinations with HGI higher than VNTR-15	Optimal combinations with highest the HGI^a^	HGI (mean ± STDEV)		
			All strains	Non-Beijing strains	Beijing strains
2 (190)	0	1-5	0.900±0.041	0.852±0.044	0.951±0.015
3 (1140)	0	1-2-5	0.948±0.027	0.917±0.030	0.973±0.014
4 (4845)	0	1-2-4-5	0.966±0.025	0.947±0.031	0.980±0.014
5 (15504)	0	1-2-3-4-5	0.974±0.022	0.961±0.027	0.984±0.012
6 (38760)	0	1-2-3-4-5-8	0.980±0.020	0.970±0.025	0.988±0.010
7 (77520)	0	1-2-3-4-5-6-8	0.984±0.017	0.977±0.022	0.991±0.005
8 (125970)	0	1-2-3-4-5-6-8-12	0.987±0.013	0.981±0.016	0.992±0.006
9 (167960)	8	**1-2-3-4-5-6-7-8-10**	0.989±0.011	0.985±0.014	0.993±0.005
10 (184756)	219	1-2-3-4-5-6-7-8-10-12	0.991±0.008	0.988±0.010	0.993±0.005
11 (167960)	1506	1-2-3-4-5-6-7-8-10-12-17	0.992±0.008	0.989±0.010	0.993±0.005
12 (125970)	4864	1-2-3-4-5-6-7-8-10-12-14-17	0.993±0.006	0.990±0.008	0.993±0.005
13 (77520)	8836	1-2-3-4-5-6-7-8-10-12-14-15-17	0.994±0.006	0.991±0.008	0.993±0.005
14 (38760)	9513	1-2-3-4-5-6-7-8-9-10-12-14-15-17	0.994±0.006	0.991±0.008	0.993±0.005
15 (15504)	6599	1-2-3-4-5-6-7-8-9-10-12-14-15-17-18	0.994±0.006	0.992±0.007	0.994±0.005
16 (4845)	3081	1-2-3-4-5-6-7-8-9-10-12-14-15-16-17-18	0.994±0.006	0.992±0.008	0.994±0.006
17 (1140)	941	1-2-3-4-5-6-7-8-9-10-12-14-15-16-17-18-20	0.995±0.006	0.992±0.008	0.994±0.006
18 (190)	181	1-2-3-4-5-6-7-8-9-10-11-12-14-15-16-17-18-20	0.995±0.006	0.992±0.008	0.994±0.006
19 (20)	20	1-2-3-4-5-6-7-8-9-10-11-12-13-14-15-16-17-18-20	0.995±0.006	0.992±0.008	0.994±0.006
20 (1)	1	1-2-3-4-5-6-7-8-9-10-11-12-13-14-15-16-17-18-19-20	0.995±0.006	0.993±0.008	0.994±0.006

a VNTR loci were numbered according to Table 1; the combination in bold indicates the optimal 9-locus VNTR set.

VNTR loci that could combine to achieve comparable resolution of *IS6110* restriction fragment length polymorphism (RFLP) were usually selected as final typing sets [16], [18]. However, such VNTR schemes usually contain large number of loci, which made them less practical, especially in developing settings. According to previous studies, the discriminatory power of standard VNTR-15 was sufficiently high for first-line typing [13], [14]. Therefore, the average HGI of VNTR-15 in six regions was set as the threshold for choosing the optimal combination in this study. A minimum of nine loci was found could achieve the discriminatory power of VNTR-15. Among all 167,960 combinations of nine loci, eight of them had a HGI equal to or slightly higher than VNTR-15. The combination, with locus QUB-11b, QUB-18, Mtub21, MIRU 26, QUB-26, Mtub04, MIRU 31, VNTR 2372 and MIRU 40, had the highest HGI, and its discriminatory powers in non-Beijing strains and Beijing strains were both slightly higher than VNTR-15. Consistently, these nine loci had the highest relative evolution rates among the 20 candidates (Figure 2), and eight of them also among the top nine loci in terms of variability. This 9-locus set (VNTR-9) also consistently showed high resolutions among all six regions (Table 3). Comparing with VNTR-15, VNTR-9 had slightly higher discriminatory powers in Guangxi, Heilongjiang and Henan, and equal or slightly lower discriminatory powers in Sichuan, Shandong and Shanghai.

**Table 3:** Discriminatory powers of VNTR-9 and VNTR-15 among six field sites and their concordance for defining clustered and unique strains.

Field sites	HGI		Number of clustered strains (clusters) defined by			% Concordance between clustered strains defined by VNTR-9 or VNTR-15	% Concordance between unique strains defined by VNTR-9 or VNTR-15
	VNTR-9	VNTR-15	VNTR-9	VNTR-15	VNTR-9 and VNTR-15		
Guangxi (n = 154)	0.994	0.993	54 (20)	53 (19)	46 (17)	86.8	93.1
Heilongjiang (n = 168)	0.968	0.966	90 (23)	97 (27)	83 (26)	85.6	90.1
Henan (n = 160)	0.992	0.987	68 (23)	69 (24)	59 (23)	85.5	92.3
Sichuan (n = 182)	0.999	0.999	39 (18)	25 (12)	18 (9)	72.0	87.9
Shandong (n = 158)	0.991	0.992	70 (23)	66 (22)	59 (23)	89.4	89.1
Shanghai (n = 345)	0.993	0.996	167 (49)	156 (52)	144 (50)	92.3	88.4
Average/total	0.9894±0.011	0.9889±0.012	488 (156)	466 (156)	409 (148)	87.8	89.7

### Concordance between VNTR-9 and VNTR-15

The standard VNTR-15/24 has been proved reliable and effective for tracing transmissions of *M. tuberculosis* through defining genotypic clusters and unique strains [31]. To evaluate the reliability of VNTR-9 for identifying transmissions, we calculated its concordances with VNTR-15 for defining both clustered and unique strains, which defined as the the prototion of isolates having clustered (or unique) VNTR-15 genotypes that also had clusted (or unique) VNTR-9 genotypes. According to VNTR-15, 466 of the1167 strains were grouped into 156 clusters, among which 34 clusters were subtyped into singletons and/or smaller clusters by two extra loci (QUB-18 and VNTR 2372) of VNTR-9. At last, VNTR-9 classified 409 of the 466 strains into 148 clusters, with a concordance of 87.8% (409/466) with VNTR-15. The remaining 57 strains were subtyped as singletons by QUB-18 and VNTR 2372, and all of them showed additional variations in three hypervariable loci (VNTR 3232, VNTR 3820 and VNTR 4120). There were 701 unique strains based on VNTR-15, and 629 of them were also classified as singletons by VNTR-9, with a concordance of 89.7% (629/701). The remaining 72 strains were assigned to 38 clusters according to VNTR-9, and 67 of them could be further differentiated as singletons by three hypervariable loci.

**Figure 3:**
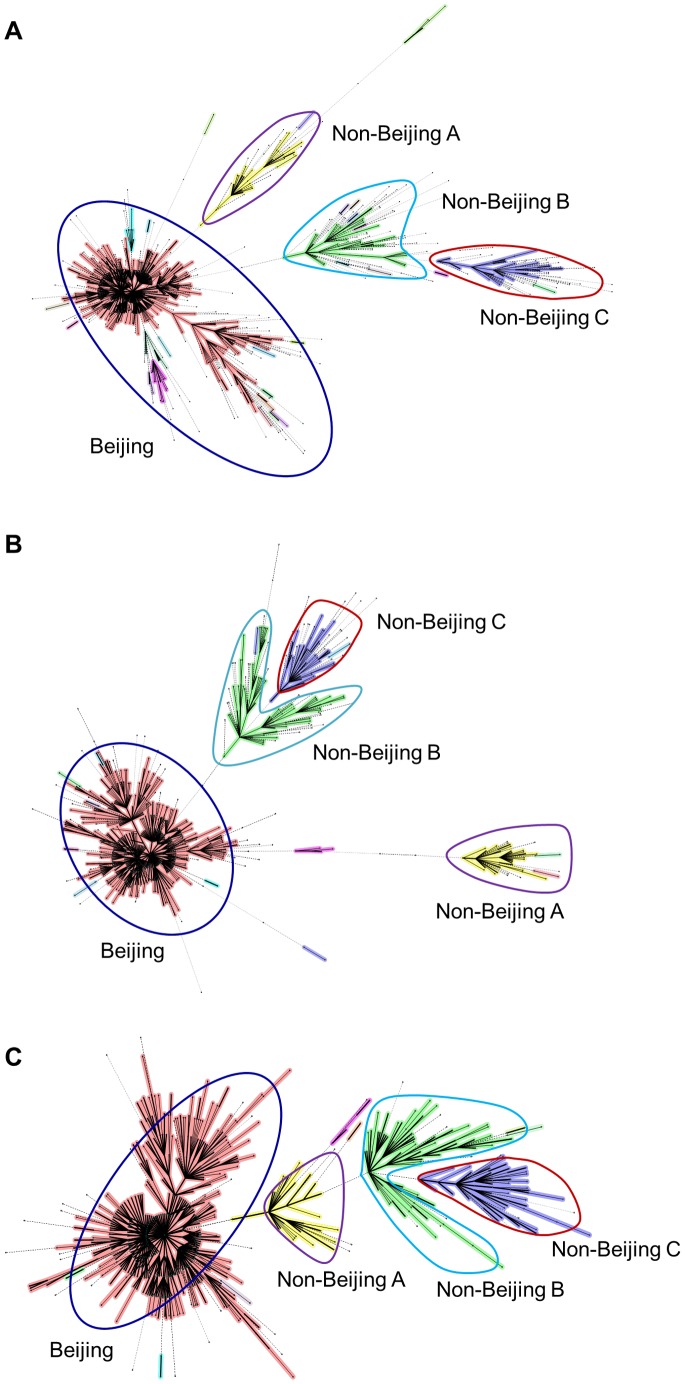
Concordance between different locus set for defining *M. tuberculosis* lineages/sublineages. Minimal spanning trees (MST) of 1167 strains based on all 20 robust VNTR loci (**A**), the standard VNTR-15 (**B**), or the optimized VNTR-9 (**C**). Clonal complexes were shaded in different colors. Circles in each MST indicated Beijing strains and three major clonal complexes of Non-Beijing strains.

We further evaluated the usefulness of VNTR-9 to predict *M. tuberculosis* lineages/sublineages. We constructed MSTs of all 1167 strains based on VNTR-20, VNTR-15 and VNTR-9 respectively. The three MSTs showed similar topologies (Figure 3). Similar to VNTR-15 and VNTR-20, the MST based on VNTR-9 successfully differentiated the four major complexes that represent Beijing strains and three sublineages of non-Beijing strains.

### Optimization of Hypervariable Locus Set for Second-line Typing

To select VNTR loci for second-line typing, we evaluated the variations of QUB-11a and three other hypervariable loci (VNTR 3232, VNTR 3820 and VNTR 4120) in clustered strains defined by VNTR-9. Among the 488 clustered stains, 441 of them had unambiguous typing results in these loci. Considerable variations within each cluster were detected in these loci, and most of them belonged to multi-locus variations (Table S5). A number of SLVs were found in three hypervariable loci (28 in VNTR 3232, 19 in VNTR 4120 and 20 in VNTR 3820), and these loci classified the 441 strains into 106 clusters (238 strains) and 103 singletons. In contrast, only two SLVs were detected in QUB-11a, and the addition of QUB-11a to the three other loci could only further differentiate two additional clusters, indicating high level of discriminatory redundancy. Since QUB-11a also associated with serious amplification problems, we excluded this locus and kept other three hypervariable loci (HV-3) as a final set for second-line typing.

**Figure 4:**
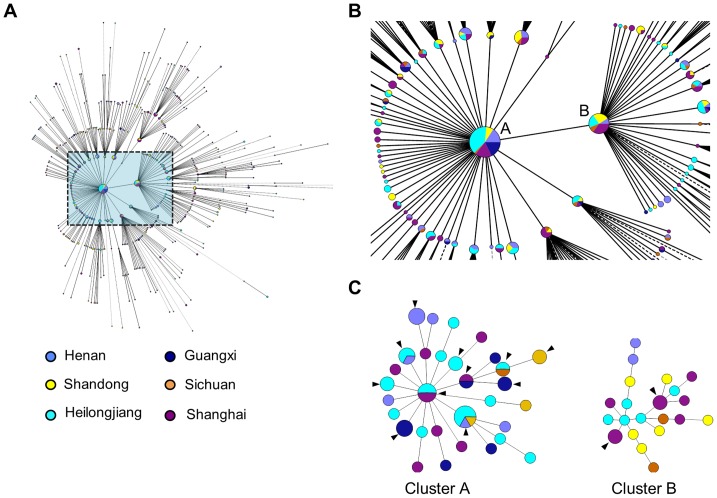
The homogeneity of “modern” Beijing strains and the differentiation of cross-regional clusters by hypervariable loci. (**A**) Minimal spanning tree (MST) of 678 “modern” Beijing strains based on 17 loci from VNTR-9 and VNTR-15; (**B**) The geographical designations for strains of the major VNTR genotype A and B, and their single-locus variants; (**C**) The MSTs of strains that belong to genotype A and B based on three hypervariable loci (QUB-3232, VNTR 3820, VNTR 4120). Black arrows indicate clusters.

The usefulness of HV-3 was further evaluated in cross-regional clusters of “modern” Beijing strains. The “modern” Beijing strains were prevalent in all field sites and accounted for 766 of all 1362 strains. A total of 678 “modern” Beijing strains had unambiguous typing results and we constructed their MST based on 17 VNTR loci that include all loci of VNTR-15 and two additional loci (VNTR 2372 and QUB-18) of VNTR-9. The MST represented a star-like network, indicating “modern” Beijing strains from different regions are highly homogenous (Figure 4A). The MST was characterized by two major genotypes (genotype A and B) in the center, which were surrounded by other minor genotypes. A number of 39 cross-regional clusters (241 strains) that contain strains from at least two different field sites were identified, and 29 of them belong to the major genotype A, B and their single locus variants (Figure 4B). Given the large geographical distance and low population motilities between field sites, these clusters less likely indicate cross-regional transmissions. According to HV-3, 147 (61.0%) of the 241 strains were differentiated as singletons. The remaining 94 strains were further classified into 40 clusters, and 29 of them only contained strains from single region. As an example, the 49 strains of cluster A and the 23 strains of cluster B were subtyped into 37 unique genotypes and 13 smaller clusters, among which eight clusters only contained strains from single region (Figure 4C).

### Clonal Stability of VNTR Loci

The clonal stability of the individual 25 loci was evaluated using 69 serial isolates obtained from 31 patients. The time spans between the initial and follow-up isolates from different patients varied from one month to 24 months (Table S6). Single locus variations were observed in two patients. Simultaneous occurrence of double alleles in VNTR 3232 was detected in the first isolates of patient 2 (with an allele of 17 or 19) or in the second isolates of patient 17 (with an allele of 9 or 11), which indicate evolution and coexistence of clonal variant strains within each patient.

Among the loci of VNTR-9, locus QUB-18 and VNTR 2372 were rarely evaluated in previous typing schemes. In this study, the HGIs of QUB-18 (0.691) and VNTR 2372 (0.496) were lower than the hypervariable loci and some loci of VNTR-15. Furthermore, the relative evolutionary rate of both loci were not extraordinarily high comparing with VNTR-15, indicating reliable clonal stabilities. We further tested the stability of QUB-18 and VNTR 2372 in 119 clusters (409 strains) defined by VNTR-15 and HV-3, which contained genetically closely related strains [13, 14). No variation of these two loci was detected in all these clusters (data not show).

## Discussion

The large TB burden and limited resource in developing countries call for applicable and highly discriminatory genotyping methods for molecular epidemiology studies. Here, by systematically evaluating 25 VNTR loci in six regions across China, we proposed an optimized 9-locus VNTR set (VNTR-9), combining with three hypervariable loci (HV-3), as the potential standard for nationwide genotyping of *M. tuberculosis* in China.

The discriminatory powers of VNTR loci, especially those of the standard VNTR-15/24, have been evaluated in many regions of China [12], [16], [18], [21]-[25], [27], [28]. Several optimized VNTR sets that contained loci with highest discriminatory powers in local regions have been proposed [16], [18], [27], [28]. These VNTR sets may be good for typing *M. tuberculosis* strains in a specific region, but their discriminatory power in other regions of China cannot be guaranteed. Furthermore, most previous studies evaluated the discriminatory powers of VNTR loci using hospital-based collection of strains [12], [22], [24], [27], [28], which is less representative in choosing loci for population-based molecular epidemiological studies. In this study, we systematically evaluated the discriminatory powers of 25 VNTR loci using population-based collections of *M. tuberculosis* strains from six field sites that cover different regions across China [4]. We found the variability for most VNTR loci varied considerably among the six sites, which could be explained by the genetic differences of *M. tuberculosis* between regions. The non-Beijing strains in China are heterogeneous, which could be classified into three distinct complexes based on their VNTR profiles (Figure 3). By contrast, the VNTR profiles of Beijing strains are highly similar and form a single large clonal complex, which, in turn, explain the high variability of VNTR loci in regions where non-Beijing strains are prevalent (Sichuan and Guangxi) and the relatively low variability in regions dominated by Beijing strains (Heilongjiang and Henan) (Table S3).

In this study, we applied a strategy to identify the optimal typing set through calculating the discriminatory power of all possible combinations. We found a minimum of nine loci could achieve the discriminatory power of standard VNTR-15. The optimal combination contains eight but not all of the nine loci with the highest HGIs. Traditionally, VNTR loci with the highest individual HGI were treated as the optimal combination. In this manner, we found a minimum of ten loci was needed to achieve comparable resolution of VNTR-15 (data not show), which highlight the limitation of using the traditional method to determine the optimal set in previous studies [15]–[18]. Among the loci of VNTR-9, seven of them are in common with VNTR-15, which enables informative comparisons between these two typing sets. On the base of these seven common loci, the addition of locus QUB-18 and VNTR 2372 could achieve comparable discriminatory power with the extra eight loci of VNTR-15. Furthermore, VNTR-9 was proved highly concordant with VNTR-15 to define both clustered and unique strains. Since VNTR-9 is six loci fewer than VNTR-15, it would largely saving the cost for genotyping, making VNTR-9 more applicable in developing countries. For the two extra loci of VNTR-9, locus QUB-18 was reported associate with instable amplification of large alleles in a previous study that included a global sample of *M. tuberculosis* strains [5]. However, we found the repeat numbers of most alleles of QUB-18 were equal or less than 10 in this study and almost all of them could be stably amplified. Similarly, QUB-18 was found with high typeability and interpretability in Beijing strains in a more recent study [19]. The discordance might thus at least in part be caused by the genetic differences of the *M. tuberculosis* strains.

Due to the prevalence of *M. tuberculosis* Beijing family in China, it is necessary to use hypervariable loci to differentiate epidemiologically unrelated strains. Recently, a consensus set of four hypervariable loci (VNTR 3232, VNTR 3820, VNTR 4120 and VNTR 1982) was proposed for second-line typing of Beijing strains following standard VNTR-24 [19]. The loci of HV-3 (VNTR 3232, VNTR 3820 and VNTR 4120) proposed in this study were all contained in the consensus set. The extra locus, VNTR 1982, which is the same loci as QUB-18, was found highly robust in terms of typeability and interpretability both in this and the recent study [19]. Therefore, it is suitable to include QUB-18 for first-line typing in this study. Considering the high variability of QUB-18 in all six areas, the inclusion of this locus would largely reduce the number of loci needed for first-line scheme. The HV-3 was proved great useful for further differentiating epidemiologically unrelated strains in this study. The 441 VNTR-9 based clustered strains were further classified into 103 singletons and 106 clusters by HV-3. Furthermore, the cross-regional clusters of “modern” Beijing strains could be mostly differentiated as singletons or smaller clusters that only contained strains from single region. However, several HV-3 based clusters still contained strains from different regions, which may indicate convergent evolutions or recent cross-regional transmissions. We also evaluated the usefulness of another locus, QUB-11a, four second-line typing. We found the inclusion of this locus only marginally improved the resolution in addition to HV-3 and, thus, we excluded it from the final typing scheme. A similar result has been observed in a recent study, in which QUB-11a was found almost fully redundant with other hypervariable loci [19]. QUB-11a was also associated with serious amplification failure in this study. This locus, together with QUB-11b and ETR A, were located closely in Rv1917c that belongs to PPE family. According to a recent study, the sequence of Rv1917c was highly polymorphic [32], which may explain the relatively low typeability of these loci.

Considering the high discriminatory powers of hypervariable loci, their clonal stabilities have been questioned [5]. In this study, we found these loci had been quite stable in serial isolates. Similar results have been observed in previous studies [14], [19], [33], indicating these loci are not extraordinarily unstable. Another concern associated with hypervariable loci is the large alleles that can not be interpreted unambiguously. The method of counting stutter peaks through a capillary electrophoresis sequencer provides an effective way to measure the repeat numbers [14]. Alternatively, we previously provide a method to minimize potential artificial variations by electrophoresing the amplicons of clustered strains defined by first-line typing in lanes close together on the same gel [13].

There are two major limitations for this study. First, two potential hypervariable loci, VNTR 3336 and QUB-15, were not evaluated in this study due to amplification failure using our PCR conditions. Amplification failure of locus QUB-15 was also reported in a previous study [34]. In a recent study, these two loci were found with low variability (0.22 for QUB-15, and 0.18 for VNTR 3336) and were not involved in any SLV events over a global panel of Beijing strains [19]. Thus, these two loci would probably not affect the determination of the typing sets in this study. Second, loci QUB-18 and VNTR 2372 from the VNTR-9 were only evaluated in this study and limited previous studies [17]–[19], and more typing data from broader areas is needed to evaluate their discriminations and robustness.

## Conclusion

Our study proposes a hierarchical VNTR typing scheme to study the transmission of *M. tuberculosis* in China. Firstly, the VNTR-9 can be used as the first-line method for large-scale genotyping. Then, the HV-3 can be used to subtype the clustered strains, based on the typing of VNTR-9. Since Beijing strains are highly prevalent in East Asia, this genotyping scheme could also be suitable for molecular epidemiology studies in other East Asian countries. The strategy to develop hierarchical VNTR typing methods that can achieve high resolution with a small number of loci could be suitable for molecular epidemiology study in other high burden countries.

## Supporting Information

Table S1 The HGI of 38 VNTR loci in different areas of East Asia and the details of different typing sets.
(DOCX)

Table S2 Locus designations and primer sequences used in this study for 27 VNTR candidate loci.
(DOCX)

Table S3 The 1362 strains from six field sites and their genetic constitutions.
(DOCX)

Table S4 The allelic distribution for each VNTR locus in non-Beijing strains and Beijing strains.
(DOCX)

Table S5 Variations of locus QUB-11a and three other hypervariable loci in 441 clustered strains defined by VNTR-9.
(DOCX)

Table S6 Stability of 25 VNTR loci in serial isolates from 31 patients.
(DOCX)
