# Supplementary material for: Development of a Hierarchical Variable-Number Tandem Repeat Typing Scheme for Mycobacterium tuberculosis in China
Source: PLoS One. 2014 Feb 25;9(2):e89726. doi: 10.1371/journal.pone.0089726 (PMC3934936; doi:10.1371/journal.pone.0089726)
Supplement: Table S1 — The HGI of 38 VNTR loci in different areas of East Asia and the details of different typing sets. (DOCX) [file pone.0089726.s001.docx]

**Table S1.** The HGI of 38 VNTR loci in different areas of East Asia and the details of different typing sets.

| **Locus** | **HGI in different settings*^a^*** | | | | | | | | | | | | | | |  | **Optimized VNTR sets*^d^*** | | | | | | | |  | **Candidate locus** |
| --- | --- | --- | --- | --- | --- | --- | --- | --- | --- | --- | --- | --- | --- | --- | --- | --- | --- | --- | --- | --- | --- | --- | --- | --- | --- | --- |
|  | **China*^b^*** | | | | | | | | | |  | **Other East Asian countries*^c^*** | | | **Median for East Asia** |  | **VNTR-24 (Europe)** | **VNTR-15 (Europe)** | **MIRU-12（Europe）** | **VNTR-12 (Japan)** | **VNTR-11 (Russia)** | **VNTR-16 (Shanghai, China)** | **VNTR-7 (Shanghai, China)** | **VNTR-12 (Hong Kong, China)** |  |  |
|  | **Taiwan (n=338)** | **Hong Kong (n= 243)** | **Beijing (n= 72)** | **Shanghai (n=189)** | **Gansu (n= 202)** | **Heilongjiang (n = 179)** | **Sichuan (n=306)** | **Tibet (n=576)** | **Jiangsu (n=246)** | **Wuhan (n=105)** |  | **Japan (n=325)** | **Russia (n=48)** | **South Korea (n=198)** |  |  |  |  |  |  |  |  |  |  |  |  |
| VNTR 3820 |  |  |  | 0.82 |  |  |  |  |  |  |  | 0.91 | 0.54 |  | **0.82** |  |  |  |  |  | **×** | **×** | **×** |  |  | **×** |
| VNTR 3232 |  | 0.80 |  |  |  |  |  |  |  |  |  | 0.93 | 0.73 |  | **0.80** |  |  |  |  |  | **×** |  |  | **×** |  | **×** |
| QUB-11b | 0.71 | 0.67 | 0.65 | 0.69 |  | 0.70 | 0.82 | 0.75 | 0.72 |  |  | 0.86 | 0.21 | 0.74 | **0.71** |  | **×** | **×** |  | **×** | **×** | **×** | **×** | **×** |  | **×** |
| VNTR 4120 |  |  |  |  |  |  |  |  |  |  |  | 0.92 | 0.37 |  | **0.65** |  |  |  |  |  | **×** |  |  |  |  | **×** |
| QUB-26 | 0.57 | 0.31 | 0.52 | 0.63 |  | 0.61 | 0.78 | 0.58 | 0.65 |  |  | 0.81 | 0.64 | 0.71 | **0.63** |  | **×** | **×** |  | **×** | **×** | **×** | **×** | **×** |  | **×** |
| MIRU 26 | 0.46 |  | 0.35 | 0.61 | 0.56 | 0.60 | 0.79 | 0.48 | 0.68 | 0.70 |  | 0.59 | 0.52 | 0.52 | **0.58** |  | **×** | **×** | **×** | **×** | **×** | **×** | **×** | **×** |  | **×** |
| Mtub21 | 0.57 |  | 0.56 | 0.54 | 0.69 | 0.40 | 0.81 | 0.54 | 0.66 |  |  | 0.73 | 0.33 | 0.61 | **0.57** |  | **×** | **×** |  | **×** | **×** | **×** | **×** |  |  | **×** |
| QUB-18 |  | 0.49 |  | 0.61 |  |  |  |  |  |  |  |  |  |  | **0.55** |  |  |  |  |  |  | **×** | **×** | **×** |  | **×** |
| QUB-11a |  | 0.51 |  | 0.54 |  |  |  |  |  |  |  | 0.84 |  |  | **0.54** |  |  |  |  |  |  | **×** | **×** | **×** |  | **×** |
| VNTR 3336 |  | 0.21 |  |  |  |  |  |  |  |  |  | 0.77 |  |  | **0.49** |  |  |  |  | **×** |  |  |  | **×** |  | **×** |
| QUB-4156 | 0.55 | 0.17 | 0.40 | 0.47 |  | 0.18 | 0.58 | 0.56 | 0.18 |  |  | 0.69 | 0.08 | 0.63 | **0.47** |  | **×** | **×** |  | **×** |  | **×** |  |  |  | **×** |
| Mtub04 | 0.65 |  | 0.31 | 0.27 |  | 0.39 | 0.75 | 0.28 | 0.47 |  |  | 0.64 | 0.00 | 0.70 | **0.43** |  | **×** | **×** |  | **×** |  | **×** |  |  |  | **×** |
| VNTR 2372 |  |  |  | 0.18 |  |  |  |  |  |  |  | 0.68 |  |  | **0.43** |  |  |  |  | **×** |  |  |  |  |  | **×** |
| VNTR 2074 |  |  |  | 0.22 |  |  |  |  |  |  |  | 0.61 |  |  | **0.42** |  |  |  |  | **×** |  | **×** |  |  |  | **×** |
| MIRU 31 | 0.59 | 0.16 | 0.17 | 0.33 | 0.37 | 0.40 | 0.67 | 0.66 |  | 0.43 |  | 0.55 | 0.16 | 0.48 | **0.41** |  | **×** | **×** | **×** | **×** | **×** | **×** |  |  |  | **×** |
| VNTR 1895 |  | 0.21 |  | 0.37 |  |  |  |  |  |  |  | 0.37 |  |  | **0.37** |  |  |  |  |  |  | **×** |  |  |  | **×** |
| ETR F |  |  |  | 0.29 |  |  |  |  | 0.34 |  |  | 0.36 |  |  | **0.34** |  |  |  |  |  |  | **×** |  |  |  | **×** |
| MIRU 40 | 0.22 |  | 0.19 | 0.15 | 0.35 | 0.29 | 0.63 | 0.25 | 0.45 | 0.41 |  | 0.47 | 0.12 | 0.53 | **0.32** |  | **×** | **×** | **×** |  | **×** |  |  | **×** |  | **×** |
| MIRU 39 | 0.19 | 0.04 | 0.12 | 0.14 | 0.10 | 0.29 | 0.75 | 0.24 | 0.39 | 0.29 |  | 0.47 | 0.00 | 0.28 | **0.24** |  | **×** |  | **×** |  |  | **×** |  | **×** |  | **×** |
| ETR A | 0.22 | 0.19 | 0.23 | 0.03 | 0.28 | 0.24 | 0.50 | 0.17 | 0.31 |  |  | 0.50 | 0.16 | 0.12 | **0.23** |  | **×** | **×** |  |  | **×** |  |  | **×** |  | **×** |
| MIRU 10 | 0.33 |  | 0.14 | 0.24 | 0.16 | 0.15 | 0.63 | 0.19 | 0.45 | 0.61 |  | 0.55 | 0.08 | 0.18 | **0.21** |  | **×** | **×** | **×** | **×** |  | **×** |  | **×** |  | **×** |
| MIRU 16 | 0.09 |  | 0.07 | 0.13 | 0.58 | 0.20 | 0.67 | 0.22 | 0.35 | 0.60 |  | 0.35 | 0.08 | 0.15 | **0.21** |  | **×** | **×** | **×** |  |  | **×** |  |  |  | **×** |
| Mtub39 | 0.21 |  | 0.17 | 0.06 | 0.12 | 0.17 | 0.69 | 0.26 | 0.39 |  |  | 0.41 | 0.00 | 0.46 | **0.21** |  | **×** | **×** |  |  |  |  |  |  |  | **×** |
| Mtub30 | 0.11 |  | 0.07 | 0.09 | 0.09 | 0.13 | 0.44 | 0.15 | 0.37 |  |  | 0.50 | 0.04 | 0.15 | **0.13** |  | **×** | **×** |  |  |  |  |  |  |  | **×** |
| QUB-15 |  | 0.13 |  | 0.03 |  |  |  |  |  |  |  | 0.58 |  |  | **0.13** |  |  |  |  | **×** |  |  |  | **×** |  | **×** |
| MIRU 04 | 0.05 | 0.07 | 0.12 | 0.06 |  | 0.21 | 0.36 | 0.10 |  | 0.15 |  | 0.19 | 0.00 | 0.05 | **0.10** |  | **×** | **×** | **×** |  |  |  |  |  |  | **×** |
| ETR C | 0.10 | 0.06 | 0.09 |  | 0.00 |  | 0.13 | 0.11 | 0.09 |  |  | 0.13 | 0.04 | 0.05 | 0.09 |  | **×** | **×** |  |  |  |  |  |  |  | **×** |
| Mtub29 | 0.01 |  | 0.12 | 0.06 |  | 0.12 | 0.08 | 0.01 |  |  |  | 0.13 | 0.09 | 0.12 | 0.09 |  | **×** |  |  |  |  |  |  |  |  |  |
| MIRU 27 | 0.02 |  | 0.01 | 0.03 | 0.10 |  | 0.62 | 0.08 | 0.24 | 0.15 |  | 0.07 | 0.00 | 0.18 | 0.08 |  | **×** |  | **×** |  |  |  |  |  |  |  |
| MIRU 20 | 0.01 |  | 0.01 | 0.06 |  |  | 0.05 | 0.41 | 0.02 | 0.24 |  | 0.08 | 0.12 | 0.11 | 0.07 |  | **×** |  | **×** |  | **×** |  |  |  |  |  |
| ETR B | 0.06 | 0.06 | 0.01 | 0.00 | 0.02 |  | 0.23 | 0.11 | 0.10 |  |  | 0.15 | 0.00 | 0.03 | 0.06 |  | **×** |  |  |  |  |  |  |  |  |  |
| Mtub34 | 0.15 |  | 0.01 | 0.09 |  |  | 0.13 | 0.03 |  |  |  | 0.04 | 0.00 | 0.07 | 0.05 |  | **×** |  |  |  |  |  |  |  |  |  |
| MIRU 23 | 0.02 |  | 0.01 | 0.06 | 0.03 |  | 0.18 | 0.03 | 0.27 | 0.34 |  | 0.34 | 0.00 | 0.03 | 0.03 |  | **×** |  | **×** |  |  |  |  |  |  |  |
| QUB-23 |  | 0.02 |  |  |  |  |  |  |  |  |  | 0.02 |  |  | 0.02 |  |  |  |  |  |  |  |  |  |  |  |
| MIRU 02 | 0.55 |  | 0.00 | 0.00 |  |  | 0.02 | 0.00 | 0.01 | 0.00 |  | 0.02 | 0.00 | 0.04 | 0.01 |  | **×** |  | **×** |  |  |  |  |  |  |  |
| QUB-1451 |  | 0.01 |  | 0.00 |  |  |  |  |  |  |  |  |  |  | 0.00 |  |  |  |  |  |  |  |  |  |  |  |
| MIRU 24 | 0.02 |  | 0.00 | 0.00 |  |  | 0.00 | 0.00 | 0.00 | 0.00 |  | 0.05 | 0.00 | 0.00 | 0.00 |  | **×** |  | **×** |  |  |  |  |  |  |  |

*^a^* n, number of isolates; boldface, loci with HGI median equal to or higher than 0.10.

*^b^* See the following references in the main text: Taiwan, 21; Hong Kong, 16; Beijing, 12; Shanghai, 18; Gansu, 22; Heilongjiang, 27; Sichuan, 28; Tibet, 27; Jiangsu, 23; Wuhan, 24. The HGIs of Hong Kong were calculated from Beijing strains. The HGIs of Sichuan were calculated from drug resistant isolates.

*^c^* See the following references in the main text: Japan, 17; Russia, 15; South Korea, 26.

*^d^* See the following references in the main text: for standard VNTR-15/24, 6; for VNTR-12 of Japan, 17; for VNTR-11 of Russia, 15; for VNTR-7/16 of Shanghai, 18; for VNTR-12 of Hong Kong, 16.
