# Supplementary material for: Development of a Hierarchical Variable-Number Tandem Repeat Typing Scheme for Mycobacterium tuberculosis in China
Source: PLoS One. 2014 Feb 25;9(2):e89726. doi: 10.1371/journal.pone.0089726 (PMC3934936; doi:10.1371/journal.pone.0089726)
Supplement: Table S2 — Locus designations and primer sequences used in this study for 27 VNTR candidate loci. (DOCX) [file pone.0089726.s002.docx]

**Table S2.** Locus designations and primer sequences used in this study for 27 VNTR candidate loci.

| **Locus** | **Alias** | **Primer pairs (5' to 3')** | **Amplicon size (bp) in H37Rv** | **Repeat no. × length (bp) in H37Rv** | **Ref.*^a^*** |
| --- | --- | --- | --- | --- | --- |
| 2163b | QUB-11b | CGTAAGGGGGATGCGGGAAATAGG | 412 | 5 × 69 + 10 | 1 |
|  |  | CGAAGTGAATGGTGGCAT |  |  |  |
| 1982 | QUB-18 | ATCGTCAGCTGCGGAATAGT | 621 | 5 × 78 + 49 | 1 |
|  |  | AATACCGGGGATATCGGTTC |  |  |  |
| 1955 | Mtub21 | AGATCCCAGTTGTCGTCGTC | 206 | 2 × 57 | 2 |
|  |  | CAACATCGCCTGGTTCTGTA |  |  |  |
| 2996 | MIRU 26 | GCGGATAGGTCTACCGTCGAAATC | 387 | 3 × 48 | 3 |
|  |  | TCCGGGTCATACAGCATGATCA |  |  |  |
| 4052 | QUB-26 | AACGCTCAGCTGTCGGAT | 708 | 5 × 111 + 24 | 1 |
|  |  | GGCCAGGTCCTTCCCGAT |  |  |  |
| 424 | Mtub04 | GTCCAGGTTGCAAGAGATGG | 269 | 2 × 51 + 30 | 2 |
|  |  | GGCATCCTCAACAACGGTAG |  |  |  |
| 3192 | MIRU 31; ETR E | CGTCGAAGAGAGCCTCATCAATCAT | 264 | 3 × 52 | 3 |
|  |  | AACCTGCTGACCGATGGCAATATC |  |  |  |
| 2372 | VNTR 2372 | ACCTCCGTTCCGATAATC | 298 | 2 × 57 + 12 | 4 |
|  |  | CAGCTTTCAGCCTCCACA |  |  |  |
| 960 | MIRU 10 | ACCGTCTTATCGGACTGCACTATCAA | 378 | 3 × 53 | 3 |
|  |  | CACCTTGGTGATCAGCTACCTCGAT |  |  |  |
| 802 | MIRU 40 | GATTCCAACAAGACGCAGATCAAGA | 276 | 1 × 50 | 3 |
|  |  | TCAGGTCTTTCTCTCACGCTCTCG |  |  |  |
| 4348 | MIRU 39 | CGGTCAAGTTCAGCACCTTCTACATC | 285 | 2 × 47 | 3 |
|  |  | GCGTCCGTACTTCCGGTTCAG |  |  |  |
| 2165 | ETR A | ATTTCGATCGGGATGTTGAT | 397 | 3 × 75 + 23 | 2 |
|  |  | TCGGTCCCATCACCTTCTTA |  |  |  |
| 4156 | QUB-4156 | TGGTCGCTACGCATCGTGTCGGCCCGT | 224 | 2 × 59 + 51 | 2 |
|  |  | TACCACCCGGGCAGTTTAC |  |  |  |
| 2401 | Mtub30 | AGTCACCTTTCCTACCACTCGTAAC | 319 | 1 × 58 + 53 | 2 |
|  |  | ATTAGTAGGGCACTAGCACCTCAAG |  |  |  |
| 3690 | Mtub39 | AATCACGGTAACTTGGGTTGTTT | 341 | 2 × 58 + 14 | 2 |
|  |  | GATGCATGTTCGACCCGTAG |  |  |  |
| 2074 | Mtub24 | AAATTCAAAGAGTTTCTCGACAGTG | 805 | 3 × 56 + 30 | 2 |
|  |  | GATCTTGAGAACCAAGATGTCCTT |  |  |  |
| 1895 | QUB-1895 | GGTGCACGGCCTCGGCTCC | 319 | 4 × 57 + 11 | 6 |
|  |  | AAGCCCCGCCGCCAATCAA |  |  |  |
| 1644 | MIRU 16 | CGGGTCCAGTCCAAGTACCTCAAT | 471 | 2 × 52 | 3 |
|  |  | GATCCTCCTGATTGCCCTGACCTA |  |  |  |
| 580 | MIRU 04; ETR D | GTCAAACAGGTCACAACGAGAGGAA | 336 | 3 × 77 | 3 |
|  |  | CCTCCACAATCAACACACTGGTCAT |  |  |  |
| 577 | ETR C | GACTTCAATGCGTTGTTGGA | 346 | 3 × 58 + 37 | 2 |
|  |  | GTCTTGACCTCCACGAGTGC |  |  |  |
| 3239 | ETR F | CTCGGTGATGGTCCGGCCGGTCAC | 476 | 2 × 79 + 66 | 5 |
|  |  | GGAAGTGCTCGACAACGCCATGCC |  |  |  |
| 2163a | QUB-11a | CCCATCCCGCTTAGCACATTCGTA | 305 | 2 × 69 + 8 | 1 |
|  |  | TTCAGGGGGGATCCGGGA |  |  |  |
| 3232 | QUB-3232 | CAGACCCGGCGTCATCAAC | 591 | 3 × 56 + 48 | 6 |
|  |  | CCAAGGGCGGCATTGTGTT |  |  |  |
| 4120 | VNTR 4120 | GTTCACCGGAGCCAACC | 447 | 2 × 57 + 23 | 4 |
|  |  | GAGGTGGTTTCGTGGTCG |  |  |  |
| 3820 | VNTR 3820 | TGCGCGGTGAATGAGACG | 444 | 3 × 57 + 26 | 4 |
|  |  | ACCTTCATCCTTGGCGAC |  |  |  |
| 3155 | QUB-15 | GGTGATCTGGTCCATCGC | 599 | 3 × 54 + 45 | 4 |
|  |  | TGTACCAGGGCCAAGACG |  |  |  |
|  |  | AGGGGTTCTCGGTCACCC | 252 |  | 1 |
|  |  | TACATTCGCGGCCAAAGG |  |  |  |
| 3336 | QUB-3336 | ATCCCCGCGGTACCCATC | 407 | 5 × 59 + 5 | 6 |
|  |  | GCCAGCGGTGTCGACTATCC |  |  |  |
|  |  | CCACCGCGATCCAGGAAT | 489 |  | 7 |
|  |  | CGGGATTCACCACGATCTC |  |  |  |
|  |  | AAACAGCACACCGGTGATTTT | 882 |  | 8 |
|  |  | TTCTACGACTTCGCAACCAAG |  |  |  |

***^a^*** See the references bellow.

1. Skuce RA, McCorry TP, McCarroll JF, Roring SM, Scott AN, et al. (2002) Discrimination of *Mycobacterium tuberculosis* complex bacteria using novel VNTR-PCR targets. Microbiology 148: 519-528.

2. Le Fleche P, Fabre M, Denoeud F, Koeck JL, Vergnaud G (2002) High resolution, on-line identification of strains from the *Mycobacterium tuberculosis* complex based on tandem repeat typing. BMC Microbiol 2: 37.

3. Kwara A, Schiro R, Cowan LS, Hyslop NE, Wiser MF, et al. (2003) Evaluation of the epidemiologic utility of secondary typing methods for differentiation of *Mycobacterium tuberculosis* isolates. J Clin Microbiol 41: 2683-2685.

4. Smittipat N, Billamas P, Palittapongarnpim M, Thong-On A, Temu MM, et al. (2005) Polymorphism of variable-number tandem repeats at multiple loci in *Mycobacterium tuberculosis*. J Clin Microbiol 43: 5034-5043.

5. Frothingham R, Meeker-O'Connell WA (1998) Genetic diversity in the *Mycobacterium tuberculosis* complex based on variable numbers of tandem DNA repeats. Microbiology 144 ( Pt 5): 1189-1196.

6. Roring S, Scott A, Brittain D, Walker I, Hewinson G, et al. (2002) Development of variable-number tandem repeat typing of *Mycobacterium bovis*: comparison of results with those obtained by using existing exact tandem repeats and spoligotyping. J Clin Microbiol 40: 2126-2133.

7. Iwamoto T, Yoshida S, Suzuki K, Tomita M, Fujiyama R, et al. (2007) Hypervariable loci that enhance the discriminatory ability of newly proposed 15-loci and 24-loci variable-number tandem repeat typing method on *Mycobacterium tuberculosis* strains predominated by the Beijing family. FEMS Microbiol Lett 270: 67-74.

8. Supply P, Allix C, Lesjean S, Cardoso-Oelemann M, Rusch-Gerdes S, et al. (2006) Proposal for standardization of optimized mycobacterial interspersed repetitive unit-variable-number tandem repeat typing of *Mycobacterium tuberculosis*. J Clin Microbiol 44: 4498-4510.
