# Supplementary material for: Development of a Hierarchical Variable-Number Tandem Repeat Typing Scheme for Mycobacterium tuberculosis in China
Source: PLoS One. 2014 Feb 25;9(2):e89726. doi: 10.1371/journal.pone.0089726 (PMC3934936; doi:10.1371/journal.pone.0089726)
Supplement: Table S3 — The 1362 strains from six field sites and their genetic constitutions. (DOCX) [file pone.0089726.s003.docx]

**Table S3.** The 1362 strains from six field sites and their genetic constitutions.

| **Field sites** | **All strains** | **Beijing strains (n, %)** | **“modern” Beijing strains (n, %)** |
| --- | --- | --- | --- |
| Sichuan | 215 | 112 (52.1%) | 65 (30.2%) |
| Guangxi | 174 | 107 (61.5%) | 58 (33.3%) |
| Shanghai | 392 | 313 (79.8%) | 233 (59.4%) |
| Shandong | 204 | 159 (77.9%) | 134 (65.7%) |
| Henan | 194 | 174 (89.7%) | 130 (67.0%) |
| Heilongjiang | 183 | 158 (86.3%) | 146 (79.8%) |
| Total | 1362 | 1023 (75.1%) | 766 (56.2%) |
