# Supplementary material for: Development of a Hierarchical Variable-Number Tandem Repeat Typing Scheme for Mycobacterium tuberculosis in China
Source: PLoS One. 2014 Feb 25;9(2):e89726. doi: 10.1371/journal.pone.0089726 (PMC3934936; doi:10.1371/journal.pone.0089726)
Supplement: Table S4 — The allelic distribution for each VNTR locus in non-Beijing strains and Beijing strains. (DOCX) [file pone.0089726.s004.docx]

**Table S4.** The allelic distribution for each VNTR locus in non-Beijing strains and Beijing strains.

| **VNTR locus** | **Subpopulations** | **Allele distribution (%)** | | | | | | | | | | | | | | | | | | | | |
| --- | --- | --- | --- | --- | --- | --- | --- | --- | --- | --- | --- | --- | --- | --- | --- | --- | --- | --- | --- | --- | --- | --- |
|  |  | **0** | **1** | **2** | **3** | **4** | **5** | **6** | **7** | **8** | **9** | **10** | **11** | **12** | **13** | **14** | **15** | **16** | **17** | **18** | **19** | **>=20** |
| QUB-11b | Beijing |  | 0.4% | 1.2% | 3.4% | 9.3% | 28.1% | 45.2% | 10.5% | 1.3% | 0.4% | 0.2% |  |  |  |  |  |  |  |  |  |  |
|  | non-Beijing |  | 20.3% | 17.3% | 24.3% | 19.6% | 9.0% | 6.0% | 2.7% | 1.0% |  |  |  |  |  |  |  |  |  |  |  |  |
| QUB 18 | Beijing | 0.2% |  | 0.3% | 2.2% | 1.1% | 1.5% | 3.3% | 5.8% | 63.3% | 4.9% | 14.7% | 1.2% | 0.6% | 0.3% | 0.2% | 0.4% |  | 0.1% |  |  |  |
|  | non-Beijing | 36.5% | 0.6% | 0.3% | 4.7% | 2.9% | 32.5% | 2.3% | 3.8% | 12.0% | 3.5% | 0.6% |  |  | 0.3% |  |  |  |  |  |  |  |
| Mtub21 | Beijing |  | 0.6% | 1.1% | 4.0% | 25.0% | 63.0% | 3.3% | 0.9% | 1.6% | 0.5% | 0.1% |  |  |  |  |  |  |  |  |  |  |
|  | non-Beijing |  | 37.6% | 7.4% | 49.4% | 3.5% | 1.5% | 0.3% |  |  |  | 0.3% |  |  |  |  |  |  |  |  |  |  |
| MIRU 26 | Beijing |  | 0.5% | 0.1% | 1.0% | 1.8% | 6.0% | 11.1% | 72.0% | 4.2% | 2.3% | 0.6% | 0.2% | 0.2% |  |  |  |  |  |  |  |  |
|  | non-Beijing |  | 20.8% | 3.2% | 2.3% | 17.8% | 47.4% | 3.5% | 4.7% |  | 0.3% |  |  |  |  |  |  |  |  |  |  |  |
| QUB-26 | Beijing |  | 0.2% | 0.6% | 0.5% | 1.2% | 1.3% | 4.8% | 14.4% | 65.3% | 8.8% | 2.4% | 0.1% | 0.4% |  |  |  |  |  |  |  |  |
|  | non-Beijing | 0.3% | 1.5% | 0.9% | 3.6% | 9.6% | 7.2% | 12.0% | 23.2% | 31.9% | 7.2% | 0.9% | 1.2% | 0.3% |  |  |  |  |  |  |  |  |
| MIRU 31 | Beijing | 0.3% |  | 0.5% | 1.2% | 9.6% | 85.7% | 2.2% | 0.4% | 0.1% |  |  |  |  |  |  |  |  |  |  |  |  |
|  | non-Beijing | 0.6% |  | 9.1% | 78.2% | 10.3% | 1.8% |  |  |  |  |  |  |  |  |  |  |  |  |  |  |  |
| Mtub04 | Beijing |  | 0.4% | 5.9% | 10.5% | 80.1% | 2.8% | 0.2% | 0.1% |  |  |  |  |  |  |  |  |  |  |  |  |  |
|  | non-Beijing |  | 6.5% | 45.9% | 24.0% | 21.6% | 2.1% |  |  |  |  |  |  |  |  |  |  |  |  |  |  |  |
| MIRU 10 | Beijing | 0.1% | 2.7% | 10.1% | 85.8% | 1.1% | 0.2% |  |  |  |  |  |  |  |  |  |  |  |  |  |  |  |
|  | non-Beijing |  | 1.2% | 73.9% | 3.0% | 5.9% | 12.8% | 2.1% | 0.6% |  | 0.3% |  |  |  |  | 0.3% |  |  |  |  |  |  |
| MIRU 39 | Beijing |  | 1.2% | 4.2% | 92.3% | 2.2% | 0.1% | 0.1% |  |  |  |  |  |  |  |  |  |  |  |  |  |  |
|  | non-Beijing |  | 1.2% | 95.0% | 3.6% | 0.3% |  |  |  |  |  |  |  |  |  |  |  |  |  |  |  |  |
| MIRU 40 | Beijing |  | 3.0% | 4.1% | 87.8% | 4.0% | 0.9% | 0.1% |  |  |  |  |  |  |  |  |  |  |  |  |  |  |
|  | non-Beijing |  | 5.9% | 50.4% | 30.6% | 11.0% | 2.1% |  |  |  |  |  |  |  |  |  |  |  |  |  |  |  |
| QUB-4156 | Beijing | 0.3% | 0.7% | 76.4% | 5.8% | 16.3% | 0.6% |  |  |  |  |  |  |  |  |  |  |  |  |  |  |  |
|  | non-Beijing | 1.5% | 6.5% | 73.2% | 18.6% | 0.3% |  |  |  |  |  |  |  |  |  |  |  |  |  |  |  |  |
| ETR A | Beijing |  | 0.1% | 0.9% | 9.5% | 89.2% | 0.2% | 0.1% |  |  |  |  |  |  |  |  |  |  |  |  |  |  |
|  | non-Beijing |  | 0.3% | 9.1% | 65.2% | 23.3% | 1.7% |  | 0.3% |  |  |  |  |  |  |  |  |  |  |  |  |  |
| Mtub30 | Beijing |  |  | 2.6% | 1.0% | 96.0% | 0.3% | 0.1% |  |  |  |  |  |  |  |  |  |  |  |  |  |  |
|  | non-Beijing |  |  | 77.6% | 0.9% | 21.5% |  |  |  |  |  |  |  |  |  |  |  |  |  |  |  |  |
| Mtub39 | Beijing |  | 1.1% | 1.2% | 92.5% | 3.7% | 1.3% | 0.3% |  |  |  |  |  |  |  |  |  |  |  |  |  |  |
|  | non-Beijing |  | 39.2% | 3.3% | 48.7% | 5.9% | 2.4% | 0.6% |  |  |  |  |  |  |  |  |  |  |  |  |  |  |
| VNTR 2074 | Beijing | 0.2% | 2.7% | 91.5% | 5.4% | 0.2% |  |  |  |  |  |  |  |  |  |  |  |  |  |  |  |  |
|  | non-Beijing | 20.5% | 11.9% | 61.3% | 5.7% | 0.6% |  |  |  |  |  |  |  |  |  |  |  |  |  |  |  |  |
| QUB-1895 | Beijing |  | 1.2% | 7.6% | 1.5% | 86.2% | 2.9% | 0.5% | 0.2% |  |  |  |  |  |  |  |  |  |  |  |  |  |
|  | non-Beijing |  | 2.7% | 7.6% | 5.1% | 81.9% | 2.1% | 0.3% |  | 0.3% |  |  |  |  |  |  |  |  |  |  |  |  |
| MIRU 16 | Beijing |  | 0.4% | 2.8% | 93.7% | 3.0% |  |  |  |  |  |  |  |  |  |  |  |  |  |  |  |  |
|  | non-Beijing |  | 18.3% | 13.9% | 62.4% | 5.0% | 0.3% |  |  |  |  |  |  |  |  |  |  |  |  |  |  |  |
| MIRU 04 | Beijing | 1.2% | 1.4% | 96.7% | 0.5% | 0.3% |  |  |  |  |  |  |  |  |  |  |  |  |  |  |  |  |
|  | non-Beijing | 0.3% | 2.7% | 59.1% | 7.7% | 27.3% | 0.9% | 0.3% | 0.6% | 0.9% |  |  |  |  |  |  |  |  |  |  |  |  |
| ETR C | Beijing |  | 0.2% | 1.7% | 0.9% | 95.4% | 1.7% | 0.2% |  |  |  |  |  |  |  |  |  |  |  |  |  |  |
|  | non-Beijing |  |  | 0.9% | 2.7% | 92.3% | 3.0% | 1.2% |  |  |  |  |  |  |  |  |  |  |  |  |  |  |
| VNTR 2372 | Beijing |  | 8.4% | 2.8% | 80.0% | 6.8% | 1.6% | 0.3% | 0.1% |  |  |  |  |  |  |  |  |  |  |  |  |  |
|  | non-Beijing |  | 2.4% | 75.4% | 20.2% | 1.2% | 0.9% |  |  |  |  |  |  |  |  |  |  |  |  |  |  |  |
| QUB-11a | Beijing |  | 0.2% | 0.2% | 0.2% | 1.6% | 18.7% | 3.1% | 5.3% | 65.3% | 4.9% |  | 0.1% |  |  |  |  |  |  |  |  |  |
|  | non-Beijing |  |  | 0.7% | 0.7% | 8.6% | 62.6% | 6.5% | 15.8% | 1.4% | 0.7% | 0.7% | 1.8% | 0.4% |  |  |  |  |  |  |  |  |
| VNTR 3820 | Beijing |  |  | 0.2% | 0.8% | 0.1% | 0.5% | 0.6% | 1.5% | 0.8% | 5.0% | 3.4% | 3.3% | 17.2% | 4.1% | 44.7% | 3.4% | 5.5% | 3.5% | 1.4% | 1.3% | 2.7% |
|  | non-Beijing |  | 3.3% | 0.3% | 39.9% | 1.8% | 28.3% | 8.0% | 6.3% | 6.3% | 3.3% |  | 0.6% | 1.5% | 0.3% | 0.3% |  |  |  |  |  | 0.0% |
| VNTR 4120 | Beijing |  |  | 0.9% | 2.7% | 3.2% | 2.8% | 5.1% | 3.4% | 15.4% | 14.8% | 34.8% | 7.4% | 3.1% | 3.0% | 0.8% | 0.7% | 0.5% | 0.2% | 0.4% | 0.1% | 0.6% |
|  | non-Beijing |  | 1.2% | 3.3% | 27.8% | 58.9% | 2.7% | 2.1% | 0.6% | 1.5% |  | 1.5% | 0.3% |  | 0.3% |  |  |  |  |  |  | 0.0% |
| VNTR 3232 | Beijing |  |  | 0.4% | 1.0% | 0.2% | 1.7% | 1.0% | 2.4% | 4.7% | 3.6% | 4.2% | 11.6% | 8.6% | 34.0% | 12.0% | 5.6% | 3.1% | 2.4% | 1.5% | 1.0% | 0.8% |
|  | non-Beijing |  | 0.6% | 0.6% | 2.1% | 10.0% | 8.8% | 19.7% | 11.8% | 18.5% | 10.0% | 5.5% | 5.5% | 1.8% | 2.1% | 0.9% | 0.3% | 0.3% |  | 0.3% | 1.2% | 0.0% |

***^a^*** The ratios were labeled with a color gradient form green to yellow. The higher of the ratio, the darker of the green; the lower, the yellower.
