# Supplementary material for: Development of a Hierarchical Variable-Number Tandem Repeat Typing Scheme for Mycobacterium tuberculosis in China
Source: PLoS One. 2014 Feb 25;9(2):e89726. doi: 10.1371/journal.pone.0089726 (PMC3934936; doi:10.1371/journal.pone.0089726)
Supplement: Table S5 — Variations of locus QUB-11a and three other hypervariable loci in 441 clustered strains defined by VNTR-9. (DOCX) [file pone.0089726.s005.docx]

**Table S5.** Variations of locus QUB-11a and three other hypervariable loci in 441 clustered strains defined by VNTR-9.

| **Field sites** | **Number of variation events*^a^*** | | | | **Number of clustered strains (clusters) defined by HV-3** |
| --- | --- | --- | --- | --- | --- |
|  | **QUB-11a** | **VNTR 3232** | **VNTR 4120** | **VNTR 3820** |  |
| Guangxi (n=50) | 2 (0) | 11 (8) | 5 (1) | 4 (2) | 21 (9) |
| Heilongjiang (n=81) | 4 (1) | 8 (8) | 13 (10) | 8 (7) | 50 (21) |
| Henan (n=56) | 1 (0) | 8 (3) | 9 (2) | 9 (4) | 33 (15) |
| Sichuan (n=31) | 4 (0) | 6 (1) | 8 (0) | 6 (2) | 10 (5) |
| Shandong (n=67) | 6 (0) | 16 (3) | 22 (2) | 12 (3) | 28 (13) |
| Shanghai (n=156) | 13 (1) | 38 (5) | 30 (4) | 27 (2) | 96 (43) |
| Total | 30 (2) | 87 (28) | 87 (19) | 66 (20) | 238 (106) |

n, number of strains in each filed site.

***^a^*** The number of variation events was calculated according to the minimal spanning network of each cluster. Values in parenthesis indicate single locus variation (SLV) events.
