# Supplementary material for: Development of a Hierarchical Variable-Number Tandem Repeat Typing Scheme for Mycobacterium tuberculosis in China
Source: PLoS One. 2014 Feb 25;9(2):e89726. doi: 10.1371/journal.pone.0089726 (PMC3934936; doi:10.1371/journal.pone.0089726)
Supplement: Table S6 — Stability of 25 VNTR loci in serial isolates from 31 patients. (DOCX) [file pone.0089726.s006.docx]

**Table S6.** Stability of 25 VNTR loci in serial isolates from 31 patients.

| **Patient ID** | **Strain ID** | **Time span (months)** | **Lineage** | **VNTR-15 specific** | | | | | | | | **Common loci for VNTR-15 and VNTR-9** | | | | | | | **VNTR-9 specific** | | **Hypervariable loci** | | | | **Other loci** | | | |
| --- | --- | --- | --- | --- | --- | --- | --- | --- | --- | --- | --- | --- | --- | --- | --- | --- | --- | --- | --- | --- | --- | --- | --- | --- | --- | --- | --- | --- |
|  |  |  |  | **MIRU 04** | **MIRU 10** | **MIRU 16** | **Mtub30** | **Mtub39** | **QUB-4156** | **ETR A** | **ETR C** | **QUB-11b** | **Mtub21** | **MIRU 26** | **QUB-26** | **Mtub04** | **MIRU 31** | **MIRU 40** | **QUB-18** | **VNTR 2372** | **Qub-11a** | **QUB-3232** | **VNTR 4120** | **VNTR 3820** | **MIRU 39** | **Mtub24** | **QUB-1895** | **ETR F** |
| 1 | P250 | 0 | Beijing | 2 | 3 | 3 | 4 | 3 | 2 | 4 | 4 | 6 | 5 | 7 | 8 | 4 | 5 | 3 | 8 | 3 | 8 | 16 | 10 | 10 | 3 | 4 | 2 | 2 |
|  | P305 | 3 | Beijing | 2 | 3 | 3 | 4 | 3 | 2 | 4 | 4 | 6 | 5 | 7 | 8 | 4 | 5 | 3 | 8 | 3 | 8 | 16 | 10 | 10 | 3 | 4 | 2 | 2 |
| 2 | P372 | 0 | Beijing | 2 | 3 | 3 | 4 | 3 | 2 | 4 | 4 | 6 | 4 | 6 | 9 | 4 | 5 | 3 | 10 | 3 | 8 | 17/19 | 9 | 14 | 3 | 2 | 3 | 2 |
|  | P388 | 1 | Beijing | 2 | 3 | 3 | 4 | 3 | 2 | 4 | 4 | 6 | 4 | 6 | 9 | 4 | 5 | 3 | 10 | 3 | 8 | 19 | 9 | 14 | 3 | 2 | 3 | 2 |
| 3 | P326 | 0 | Beijing | 2 | 3 | 3 | 4 | 3 | 2 | 4 | 4 | 6 | 5 | 8 | 8 | 4 | 4 | 3 | 7 | 3 | 8 | 9 | 9 | 11 | 3 | 4 | 2 | 2 |
|  | P377 | 1 | Beijing | 2 | 3 | 3 | 4 | 3 | 2 | 4 | 4 | 6 | 5 | 8 | 8 | 4 | 4 | 3 | 7 | 3 | 8 | 9 | 9 | 11 | 3 | 4 | 2 | 2 |
| 4 | P368 | 0 | non-Beijing | 3 | 2 | 3 | 2 | 1 | 3 | 4 | 4 | 5 | 1 | 4 | 8 | 3 | 2 | 3 | 0 | 2 | 5 | 5 | 2 | 3 | 2 | 4 | 2 | 2 |
|  | P395 | 3 | non-Beijing | 3 | 2 | 3 | 2 | 1 | 3 | 4 | 4 | 5 | 1 | 4 | 8 | 3 | 2 | 3 | 0 | 2 | 5 | 5 | 2 | 3 | 2 | 4 | 2 | 2 |
| 5 | P337 | 0 | Beijing | 2 | 3 | 3 | 4 | 3 | 2 | 4 | 4 | 6 | 5 | 7 | 8 | 4 | 5 | 3 | 8 | 3 | 8 | 13 | 10 | 15 | 3 | 4 | 2 | 2 |
|  | P371 | 1 | Beijing | 2 | 3 | 3 | 4 | 3 | 2 | 4 | 4 | 6 | 5 | 7 | 8 | 4 | 5 | 3 | 8 | 3 | 8 | 13 | 10 | 15 | 3 | 4 | 2 | 2 |
| 6 | P399 | 0 | Beijing | 2 | 3 | 3 | 4 | 3 | 2 | 4 | 4 | 6 | 5 | 7 | 7 | 4 | 5 | 3 | 6 | 3 | 8 | 13 | 11 | 15 | 3 | 4 | 2 | 2 |
|  | P403 | 1 | Beijing | 2 | 3 | 3 | 4 | 3 | 2 | 4 | 4 | 6 | 5 | 7 | 7 | 4 | 5 | 3 | 6 | 3 | 8 | 13 | 11 | 15 | 3 | 4 | 2 | 2 |
| 7 | P315 | 0 | non-Beijing | 3 | 2 | 3 | 2 | 1 | 2 | N | 4 | N | 1 | 5 | 8 | 4 | 4 | 3 | 0 | 2 | N | 6 | 4 | 3 | 2 | 4 | 2 | 2 |
|  | P325 | 1 | non-Beijing | 3 | 2 | 3 | 2 | 1 | 2 | N | 4 | N | 1 | 5 | 8 | 4 | 4 | 3 | 0 | 2 | N | 6 | 4 | 3 | 2 | 4 | 2 | 2 |
| 8 | P381 | 0 | Beijing | 2 | 3 | 3 | 4 | 3 | 4 | 4 | 4 | 7 | 4 | 3 | 8 | 4 | 5 | 3 | 11 | 3 | 7 | 20 | 10 | 12 | 3 | 4 | 2 | 2 |
|  | P396 | 1 | Beijing | 2 | 3 | 3 | 4 | 3 | 4 | 4 | 4 | 7 | 4 | 3 | 8 | 4 | 5 | 3 | 11 | 3 | 7 | 20 | 10 | 12 | 3 | 4 | 2 | 2 |
| 9 | P351 | 0 | non-Beijing | 3 | 2 | 4 | 2 | 1 | 2 | 4 | 4 | 7 | 1 | 5 | 7 | 4 | 3 | 2 | 0 | 2 | 4 | 6 | 3 | 3 | 2 | 1 | 2 | 2 |
|  | P360 | 3 | non-Beijing | 3 | 2 | 4 | 2 | 1 | 2 | 4 | 4 | 7 | 1 | 5 | 7 | 4 | 3 | 2 | 0 | 2 | 4 | 6 | 3 | 3 | 2 | 1 | 2 | 2 |
| 10 | P331 | 0 | Beijing | 2 | 3 | 3 | 4 | 3 | 2 | 4 | 4 | 6 | 5 | 5 | 8 | 4 | 6 | 2 | 8 | 3 | 8 | 6 | 6 | 14 | 3 | 4 | 2 | 2 |
|  | P373 | 2 | Beijing | 2 | 3 | 3 | 4 | 3 | 2 | 4 | 4 | 6 | 5 | 5 | 8 | 4 | 6 | 2 | 8 | 3 | 8 | 6 | 6 | 14 | 3 | 4 | 2 | 2 |
| 11 | P328 | 0 | Beijing | 2 | 3 | 3 | 4 | 3 | 4 | 4 | 4 | 6 | 4 | 7 | 7 | 2 | 5 | 3 | 10 | 4 | 4 | 5 | 6 | 13 | 3 | 2 | 3 | 2 |
|  | P349 | 1 | Beijing | 2 | 3 | 3 | 4 | 3 | 4 | 4 | 4 | 6 | 4 | 7 | 7 | 2 | 5 | 3 | 10 | 4 | 4 | 5 | 6 | 13 | 3 | 2 | 3 | 2 |
| 12 | P236 | 0 | non-Beijing | 4 | 2 | 3 | 2 | 1 | 2 | 3 | 4 | 3 | 1 | 5 | 8 | 3 | 3 | 2 | 0 | 2 | 4 | 5 | 4 | 3 | 2 | 4 | 2 | 2 |
|  | P255 | 1 | non-Beijing | 4 | 2 | 3 | 2 | 1 | 2 | 3 | 4 | 3 | 1 | 5 | 8 | 3 | 3 | 2 | 0 | 2 | 4 | 5 | 4 | 3 | 2 | 4 | 2 | 2 |
| 13 | P362 | 0 | Beijing | 2 | 3 | 3 | 4 | 3 | 2 | 4 | 4 | 7 | 8 | 7 | 8 | 4 | 5 | 3 | 6 | 3 | 8 | 12 | 10 | 12 | 3 | 4 | 2 | 2 |
|  | P385 | 1 | Beijing | 2 | 3 | 3 | 4 | 3 | 2 | 4 | 4 | 7 | 8 | 7 | 8 | 4 | 5 | 3 | 6 | 3 | 8 | 12 | 10 | 12 | 3 | 4 | 2 | 2 |
| 14 | P287 | 0 | Beijing | 2 | 3 | 3 | 4 | 3 | 2 | 4 | 4 | 6 | 5 | 6 | 8 | 2 | 5 | 3 | 8 | 3 | 8 | 15 | 10 | 14 | 3 | 4 | 2 | 2 |
|  | P298 | 1 | Beijing | 2 | 3 | 3 | 4 | 3 | 2 | 4 | 4 | 6 | 5 | 6 | 8 | 2 | 5 | 3 | 8 | 3 | 8 | 15 | 10 | 14 | 3 | 4 | 2 | 2 |
| 15 | P390 | 0 | non-Beijing | 4 | 2 | 3 | 2 | 1 | 2 | 4 | 4 | 6 | 1 | 3 | 8 | 5 | 3 | 2 | 0 | 2 | 4 | 10 | 3 | 3 | 2 | 2 | 2 | 2 |
|  | P445 | 6 | non-Beijing | 4 | 2 | 3 | 2 | 1 | 2 | 4 | 4 | 6 | 1 | 3 | 8 | 5 | 3 | 2 | 0 | 2 | 4 | 10 | 3 | 3 | 2 | 2 | 2 | 2 |
| 16 | P290 | 0 | Beijing | 2 | 3 | 3 | 4 | 3 | 2 | 4 | 4 | 6 | 5 | 7 | 8 | 4 | 5 | 3 | 8 | 3 | 7 | 12 | 10 | 14 | 3 | 3 | 2 | 2 |
|  | P313 | 2 | Beijing | 2 | 3 | 3 | 4 | 3 | 2 | 4 | 4 | 6 | 5 | 7 | 8 | 4 | 5 | 3 | 8 | 3 | 7 | 12 | 10 | 14 | 3 | 3 | 2 | 2 |
| 17 | P334 | 0 | non-Beijing | 2 | 6 | 2 | 4 | 3 | 1 | 3 | 4 | 2 | 2 | 1 | 8 | 2 | 4 | 3 | 9 | 4 | 7 | 11 | 3 | 1 | 2 | 3 | 0 | 1.5 |
|  | P344 | 1 | non-Beijing | 2 | 6 | 2 | 4 | 3 | 1 | 3 | 4 | 2 | 2 | 1 | 8 | 2 | 4 | 3 | 9 | 4 | 7 | 9/11 | 3 | 1 | 2 | 3 | 0 | 1.5 |
| 18 | P231 | 0 | Beijing | 2 | 3 | 3 | 4 | 3 | 2 | 4 | 4 | 5 | 5 | 7 | 7 | 4 | 6 | 3 | 8 | 3 | 1 | 14 | 9 | 17 | 3 | 4 | 2 | 2 |
|  | P297 | 5 | Beijing | 2 | 3 | 3 | 4 | 3 | 2 | 4 | 4 | 5 | 5 | 7 | 7 | 4 | 6 | 3 | 8 | 3 | 1 | 14 | 9 | 17 | 3 | 4 | 2 | 2 |
|  | P311 | 6 | Beijing | 2 | 3 | 3 | 4 | 3 | 2 | 4 | 4 | 5 | 5 | 7 | 7 | 4 | 6 | 3 | 8 | 3 | 1 | 14 | 9 | 17 | 3 | 4 | 2 | 2 |
| 19 | P408 | 0 | Beijing | 2 | 3 | 3 | 4 | 3 | 2 | 4 | 4 | 6 | 5 | 5 | 8 | 4 | 5 | 1 | 8 | 3 | 8 | 8 | 6 | 14 | 3 | 4 | 2 | 2 |
|  | P453 | 5 | Beijing | 2 | 3 | 3 | 4 | 3 | 2 | 4 | 4 | 6 | 5 | 5 | 8 | 4 | 5 | 1 | 8 | 3 | 8 | 8 | 6 | 14 | 3 | 4 | 2 | 2 |
| 20 | P069 | 0 | Beijing | 2 | 3 | 3 | 4 | 3 | 2 | 4 | 4 | 6 | 5 | 7 | 8 | 4 | 5 | 3 | 8 | 3 | 8 | 13 | 8 | 14 | 3 | 4 | 2 | 1.5 |
|  | P262 | 15 | Beijing | 2 | 3 | 3 | 4 | 3 | 2 | 4 | 4 | 6 | 5 | 7 | 8 | 4 | 5 | 3 | 8 | 3 | 8 | 13 | 8 | 14 | 3 | 4 | 2 | 1.5 |
|  | P369 | 24 | Beijing | 2 | 3 | 3 | 4 | 3 | 2 | 4 | 4 | 6 | 5 | 7 | 8 | 4 | 5 | 3 | 8 | 3 | 8 | 13 | 8 | 14 | 3 | 4 | 2 | 1.5 |
| 21 | P253 | 0 | non-Beijing | 2 | 2 | 1 | 2 | 3 | 1 | 3 | 4 | 2 | 2 | 5 | 7 | 2 | 3 | 4 | 3 | 2 | N | 4 | 4 | 5 | 2 | 4 | 1 | 1 |
|  | P280 | 1 | non-Beijing | 2 | 2 | 1 | 2 | 3 | 1 | 3 | 4 | 2 | 2 | 5 | 7 | 2 | 3 | 4 | 3 | 2 | N | 4 | 4 | 5 | 2 | 4 | 1 | 1 |
|  | P292 | 2 | non-Beijing | 2 | 2 | 1 | 2 | 3 | 1 | 3 | 4 | 2 | 2 | 5 | 7 | 2 | 3 | 4 | 3 | 2 | N | 4 | 4 | 5 | 2 | 4 | 1 | 1 |
| 22 | P181 | 0 | Beijing | 2 | 3 | 3 | 2 | 3 | 4 | 4 | 4 | 7 | 4 | 7 | 4 | 4 | 5 | 3 | 12 | 3 | 7 | 12 | 6 | 14 | 3 | 2 | 3 | 2 |
|  | P352 | 10 | Beijing | 2 | 3 | 3 | 2 | 3 | 4 | 4 | 4 | 7 | 4 | 7 | 4 | 4 | 5 | 3 | 12 | 3 | 7 | 12 | 6 | 14 | 3 | 2 | 3 | 2 |
| 23 | P309 | 0 | Beijing | 2 | 3 | 3 | 4 | 3 | 2 | 3 | 4 | 5 | 5 | 6 | 8 | 4 | 5 | 3 | 8 | 3 | 5 | 13 | 12 | 14 | 3 | 4 | 2 | 2 |
|  | P329 | 3 | Beijing | 2 | 3 | 3 | 4 | 3 | 2 | 3 | 4 | 5 | 5 | 6 | 8 | 4 | 5 | 3 | 8 | 3 | 5 | 13 | 12 | 14 | 3 | 4 | 2 | 2 |
|  | P406 | 9 | Beijing | 2 | 3 | 3 | 4 | 3 | 2 | 3 | 4 | 5 | 5 | 6 | 8 | 4 | 5 | 3 | 8 | 3 | 5 | 13 | 12 | 14 | 3 | 4 | 2 | 2 |
| 24 | P374 | 0 | Beijing | 2 | 3 | 3 | 4 | 3 | 2 | 4 | 4 | 6 | 5 | 5 | 8 | 4 | 5 | 3 | 8 | 3 | 8 | 10 | 6 | 14 | 3 | 4 | 2 | 2 |
|  | P346 | 1 | Beijing | 2 | 3 | 3 | 4 | 3 | 2 | 4 | 4 | 6 | 5 | 5 | 8 | 4 | 5 | 3 | 8 | 3 | 8 | 10 | 6 | 14 | 3 | 4 | 2 | 2 |
| 25 | P219 | 0 | Beijing | 2 | 3 | 3 | 4 | 3 | 2 | 4 | 4 | 5 | 4 | 6 | 7 | 4 | 5 | 3 | 9 | 3 | 8 | 16 | 9 | 14 | 3 | 2 | 1 | 2 |
|  | P409 | 17 | Beijing | 2 | 3 | 3 | 4 | 3 | 2 | 4 | 4 | 5 | 4 | 6 | 7 | 4 | 5 | 3 | 9 | 3 | 8 | 16 | 9 | 14 | 3 | 2 | 1 | 2 |
| 26 | P348 | 0 | non-Beijing | 2 | 2 | 3 | 2 | 3 | 2 | 3 | 4 | 3 | 2 | 5 | 7 | 3 | 3 | 3 | 5 | 2 | 5 | 13 | 5 | 5 | 2 | 2 | 2 | 0 |
|  | P342 | 1 | non-Beijing | 2 | 2 | 3 | 2 | 3 | 2 | 3 | 4 | 3 | 2 | 5 | 7 | 3 | 3 | 3 | 5 | 2 | 5 | 13 | 5 | 5 | 2 | 2 | 2 | 0 |
|  | P361 | 2 | non-Beijing | 2 | 2 | 3 | 2 | 3 | 2 | 3 | 4 | 3 | 2 | 5 | 7 | 3 | 3 | 3 | 5 | 2 | 5 | 13 | 5 | 5 | 2 | 2 | 2 | 0 |
|  | P380 | 3 | non-Beijing | 2 | 2 | 3 | 2 | 3 | 2 | 3 | 4 | 3 | 2 | 5 | 7 | 3 | 3 | 3 | 5 | 2 | 5 | 13 | 5 | 5 | 2 | 2 | 2 | 0 |
| 27 | P264 | 0 | Beijing | 2 | 3 | 3 | 4 | 3 | 4 | 4 | 5 | 7 | 4 | 5 | 8 | 4 | 5 | 3 | 9 | 3 | 9 | 9 | 9 | 12 | 3 | 4 | 2 | 2 |
|  | P271 | 1 | Beijing | 2 | 3 | 3 | 4 | 3 | 4 | 4 | 5 | 7 | 4 | 5 | 8 | 4 | 5 | 3 | 9 | 3 | 9 | 9 | 9 | 12 | 3 | 4 | 2 | 2 |
| 28 | P102 | 0 | Beijing | 2 | 3 | 3 | 4 | 3 | 2 | 4 | 5 | 5 | 5 | 7 | 4 | 4 | 5 | 3 | 8 | 3 | 1 | 13 | 11 | 14 | 3 | 4 | 2 | 2 |
|  | P296 | 15 | Beijing | 2 | 3 | 3 | 4 | 3 | 2 | 4 | 5 | 5 | 5 | 7 | 4 | 4 | 5 | 3 | 8 | 3 | 1 | 13 | 11 | 14 | 3 | 4 | 2 | 2 |
|  | P336 | 19 | Beijing | 2 | 3 | 3 | 4 | 3 | 2 | 4 | 5 | 5 | 5 | 7 | 4 | 4 | 5 | 3 | 8 | 3 | 1 | 13 | 11 | 14 | 3 | 4 | 2 | 2 |
| 29 | P324 | 0 | Beijing | 2 | 3 | 3 | 4 | 3 | 3 | 4 | 4 | 6 | 3 | 7 | 10 | 3 | 5 | 1 | 10 | 3 | 5 | 9 | 8 | 9 | 3 | 4 | 2 | 1.5 |
|  | P378 | 1 | Beijing | 2 | 3 | 3 | 4 | 3 | 3 | 4 | 4 | 6 | 3 | 7 | 10 | 3 | 5 | 1 | 10 | 3 | 5 | 9 | 8 | 9 | 3 | 4 | 2 | 1.5 |
| 30 | P205 | 0 | Beijing | 1 | 3 | 3 | 4 | 3 | 4 | 4 | 4 | 5 | 4 | 7 | 8 | 4 | 5 | 3 | 10 | 3 | 7 | 15 | 8 | 16 | 3 | 4 | 2 | 2 |
|  | P229 | 2 | Beijing | 1 | 3 | 3 | 4 | 3 | 4 | 4 | 4 | 5 | 4 | 7 | 8 | 4 | 5 | 3 | 10 | 3 | 7 | 15 | 8 | 16 | 3 | 4 | 2 | 2 |
| 31 | P299 | 0 | Beijing | 2 | 3 | 3 | 4 | 3 | 2 | 4 | 4 | 6 | 5 | 7 | 8 | 4 | 5 | 3 | 6 | 3 | 8 | 15 | 10 | 14 | 3 | 4 | 2 | 2 |
|  | P319 | 1 | Beijing | 2 | 3 | 3 | 4 | 3 | 2 | 4 | 4 | 6 | 5 | 7 | 8 | 4 | 5 | 3 | 6 | 3 | 8 | 15 | 10 | 14 | 3 | 4 | 2 | 2 |

N, no amplification.
